# Host and geographically related genetic variation in species of *Cloacina* (Nematoda: Strongyloidea) from western and eastern grey kangaroos, *Macropus fuliginosus* and *M. giganteus* (Marsupialia: Macropodidae)

**DOI:** 10.1017/S0031182024001458

**Published:** 2024-12

**Authors:** Shane Gerald Middleton, Anson Koehler, Ian Beveridge

**Affiliations:** Department of Veterinary Biosciences, University of Melbourne, Melbourne, Victoria, Australia

**Keywords:** *Cloacina*, co-speciation, ITS sequence data, kangaroos, *Macropus*, nematodes

## Abstract

Specimens of *Cloacina artemis*, *C. expansa*, *C. hera*, *C. hermes*, *C. hestia*, *C. magnipapillata*, *C. obtusa* and *C. selene*, which occur in both of the closely related species of grey kangaroos, *Macropus fuliginosus* and *M. giganteus*, were found to differ genetically based on sequence data derived from the internal transcribed spacers (ITS-1, ITS-2) of ribosomal DNA. The extent of differences varied from a single base pair in *C. expansa*, to 32 in *C. hestia*. In the case of *C. hera*, *C. hestia* and *C. magnipapillata*, separate genotypes were found in *M. fuliginosus* and *M. giganteus*. With *C. artemis*, *C. expansa*, *C. obtusa* and *C. selene*, nematode genotypes did not correspond with host distributions. In *C. hermes*, two genotypes were detected but they were not related phylogenetically. The data provide evidence suggestive of genetic differentiation in most of the nematode species potentially associated with host speciation, but with differing degrees of genetic differentiation and different associations with the two host species possibly related to changes in the geographical distribution of the hosts over time.

## Graphical abstract

**Figure:**
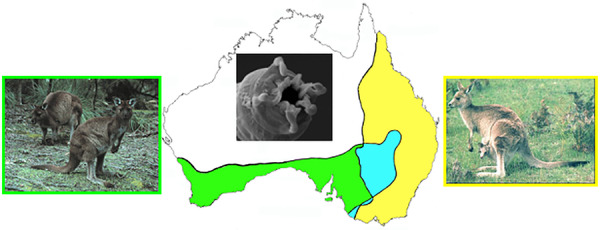

## Introduction

Co-speciation, in which speciation of a host initiates speciation in the parasites of the 2 new host species is a well-recognised paradigm of parasite evolution (Brooks and MacLennan, 1993). Colonisation, whereby ecological processes result in a parasite infecting a new sympatric but not necessarily closely related host species and subsequently diverging genetically from the founding population is an alternative mode of speciation in parasites (for definitions see Hoberg, 2005). Although initially propounded based on morphological studies, the advent of molecular techniques has allowed much closer scrutiny of this phenomenon, with numerous studies in which the speciation of parasites and their hosts has been examined initially using multi-locus allozyme electrophoresis, or later, DNA sequence data. These studies have resulted in a recent consensus that colonisation is the more frequent mode of parasite evolution (Nylin *et al*., 2017). The colonisation hypothesis has been the dominant hypothesis in recent studies of the cloacinine nematodes (Strongylida: Chabertiidae), a sub-family of nematodes found in the sacculated fore-stomachs of Australian kangaroos and wallabies (Marsupialia: Macropodidae). Examination of the genera *Rugopharynx, Pharyngostrongylus, Cyclostrongylus, Cloacina, Labiosimplex* and *Labiomultiplex* using molecular sequence data found few examples of co-speciation with most relationships better explained by colonisation events (Chilton *et al*., 2011, 2016*a*, 2016*b*, 2016*c*, 2017), but with some evidence of within-host speciation in the case of the genus *Cloacina* (Chilton *et al*., 2017).

Most such studies have focussed on host speciation events that have occurred in the distant past between hosts which may not be closely related, rather than on host-parasite systems in more recent phases of speciation. Exceptions are studies of anoplocephalid cestodes in Arctic rodents undergoing active speciation since the Pleistocene (Haukisalmi *et al*., 2001; Wickström *et al*., 2001, 2003) and the studies of Barker and Close (1990) and of Chilton *et al*. (2009) on the boopid lice and cloacinid nematode parasites, respectively, of closely related species of rock wallabies in Australia. However, the boopid study was based on parasite morphology only.

In this study, the effects of a single relatively recent host speciation event in grey kangaroos were examined on a series of their gastric-inhabiting strongylid nematodes. The eastern (*Macropus giganteus*) and western (*M. fuliginosus*) grey kangaroos are thought to have diverged relatively recently, possibly within the last 2 million years (Meredith *et al*., 2008), in the south-eastern and south-western corners of Australia, probably due to aridification, a marine incursion or some other geographical impediment along the southern Australian coastline (Kirsch and Poole, 1972; Maynes, 1989). This pattern of vicariant speciation is known for a variety of animals in southern Australia (Heatwole, 1987) including oxyurid nematodes of frogs (Inglis, 1971).

The 2 species of grey kangaroos are very similar morphologically and for many years were considered to constitute a single variable species (Kirsch and Poole, 1972). Subsequent to speciation, it is believed that *M. fuliginosus* migrated eastwards (Maynes, 1989) so that it is now sympatric with *M. giganteus* in south-western Queensland, western New South Wales, north-western Victoria and south-eastern South Australia (Caughley *et al*., 1984). The two species hybridise in captivity (Kirsch and Poole, 1972; Poole and Catling, 1974) and possible hybrids have been seen rarely in wild populations (Coulson and Coulson, 2001). However, a molecular study found that genetic introgression between the two species was widespread in the zone of kangaroo sympatry (Neaves *et al*., 2010). In addition, an insular population of *M. giganteus* occurs in Tasmania and a morphologically distinctive sub-species of *M. fuliginosus* occurs on Kangaroo Island, off South Australia, both islands having been separated from the mainland for about 10 000 years (Jennings, 1971; Dailey *et al*., 1989). The geographical isolation of island populations also provides opportunities for speciation of both hosts and parasites. The 2 species of kangaroos are closely related phylogenetically (Meredith *et al*., 2008) and the phylogeography of both species has been studied. *Macropus giganteus* is relatively uniform genetically over its range in eastern Australia showing a decline in heterozygosity in northern regions of its geographical range (Zenger *et al*., 2003). By contrast, within *M. fuliginosus*, there are several genetic groupings, some of which are linked to geographical barriers (Neaves *et al*., 2009).

The most speciose genus of nematodes found in macropodid marsupials is the strongylid genus *Cloacina*, with more than 140 species described to date (Beveridge and Smales, 2022). All occur in the sacculated fore-stomachs of their hosts, usually with multiple species of nematodes in each macropodid host species (Beveridge *et al*., 2002), and host specificity within the genus is high (Beveridge *et al*., 2002). Limited molecular data are available for a number of species (Beveridge *et al*., 2019). A series of species, *C. artemis*, *C. expansa*, *C. hermes*, *C. hera*, *C. herceus*, *C. hestia*, *C. magnipapillata*, *C. obtusa*, *C. selene*, occur in both *M. fuliginosus* and *M. giganteus*.

These species have been relatively well studied with collections available across much of the geographical range of the two host species (Beveridge, 1998). No morphological differences have been detected morphologically between populations of nematodes from the two host species apart from the observation by Beveridge (1998) of a difference in mean spicule length in populations of *C. magnipapillata* from the two host species. Chilton *et al*. (2004) examined populations of *C. obtusa* using multilocus allozyme electrophoresis and found that those from *M. fuliginosus* differed at 5% of 20 loci in specimens collected from *M. giganteus*. However, these were not considered evidence for distinct species, rather it was concluded that in this case, the nematode had failed to speciate following speciation of the hosts. By contrast, in the case of the nematode confamilial genera *Macropostrongyloides* and *Paramacropostrongylus*, allozyme and subsequent DNA sequence data have shown that in each case, a pair of closely related parasite species occurs in *M. fuliginosus* and *M. giganteus* respectively (Beveridge *et al*., 1993; Chilton *et al*., 1993; Sukee *et al*., 2020). In the case of *Paramacropostrongylus*, the two nematode species were shown to hybridise within the zone of kangaroo sympatry (Chilton *et al*., 1997). Thus, there is evidence to indicate that speciation in the grey kangaroos has already resulted in speciation in some of its parasites.

In this study, representatives of the suite of species of *Cloacina* collected from both species of kangaroos throughout the relevant host geographic ranges were compared using the first and second internal transcribed spacers (ITS-1 and ITS-2) of ribosomal DNA as markers. These DNA sequences show little intra-specific variation across wide geographic ranges within strongylid nematodes yet show distinct differences between species (Chilton, 2004; Gasser *et al*., 2009) and are considered to be useful in ‘species prospecting’ (Nadler and Pérez-Ponce de León, 2011). Our hypothesis was that speciation in the grey kangaroos may have at least initiated genetic differentiation in the range of *Cloacina* species they harbour since this has occurred in some other nematodes found in the same host species. These and other species of *Cloacina* may occur in their hosts across wide geographical ranges and the possibility that geographical distances together with genetic drift leading to genetic differentiation within the same widely distributed host species has been addressed by Shuttleworth *et al*. (2014, 2016) for the species of *Cloacina* found in the swamp wallaby, *Wallabia bicolor*, and the wallaroo, *Osphranter robustus*, respectively. Consequently, one species, *C. herceus,* which occurs essentially in *M. giganteus*, but across an extensive geographical range, was included in this study to test this particular hypothesis.

## Materials and methods

Kangaroos were either collected opportunistically as fresh road-kills or were obtained from professional shooters. In a few instances, carcases had been frozen prior to examination for parasites. Nematodes were washed in saline if alive or water if thawed from frozen carcases and were (re-)frozen in liquid nitrogen and subsequently stored at −80°C. Additional samples of nematodes from each host were fixed in Berland's fluid (glacial acetic acid and formalin) (Gibson, 1979) for morphological examination and use in confirming identifications.

Frozen nematodes were thawed, the head and tail removed, cleared in lactophenol and stored in ethanol as voucher specimens, with the mid-body region being used for genetic analyses. Nematodes were identified following the key of Beveridge (1998). Voucher specimens of all nematodes studied have been deposited in the Australian Helminthological Collection in the South Australian Museum, Adelaide (SAM); registration numbers 49135-49207. Host nomenclature follows Jackson and Groves (2015). Host distributions are from van Dyck and Strahan (2008). Abbreviations of Australian state names used in the results section and in the Tables are: NSW, New South Wales; Qld, Queensland; SA, South Australia; Tas, Tasmania; Vic, Victoria and WA, Western Australia.

Genomic DNA was isolated from individual nematodes using a small-scale sodium-dodecyl-sulphate/proteinase K extraction procedure (Gasser *et al*., 1993), followed by mini-column (Wizard™ Clean-Up, Promega, Madison, WI, USA) purification. Nematode DNA was initially examined using polymerase chain reaction (PCR). The ITS-1 region was amplified by PCR using primers NC16 (forward; 5′-AGTTCAATCGCAATGGCTT-3′) and NC13R (reverse; 5′-GCTGCGTTCTTCATCGAT-3′) while the ITS-2 region was amplified using NC1 (forward; 5′-ACGTCTGGTTCAGGGTTGTT-3′) and NC2 (reverse; 5′-TTAGTTTCTTTTCCTCCGCT-3′) (Chilton, 2004). PCR was performed in a 50 *μ*L volume for 30 cycles at 94°C for 30 s (denaturation), 55°C for 30 s (annealing) and 72°C for 30 s (extension), followed by one cycle at 72°C for 5 min (final extension). Negative (no-DNA) and known positive controls were included in each set of reactions. Amplicons were examined on ethidium bromide-stained 1.5% agarose-TBE gels using Φ X174-*Hae*III size markers. Amplicons then underwent single-strand conformation polymorphism (SSCP) analysis as described previously (Gasser *et al*., 2006) and the number of specimens with each sequence profile was calculated. Subsequently, samples with variable band profiles in the SSCP analysis were sequenced in both orientations using the same primers following purification using mini-columns (Wizard™ PCR-Prep, Promega).

The 5′- and 3′- ends of the sequences were established based on comparison with other strongylid nematodes (Chilton *et al*., 1997; Newton *et al*., 1998). The sequences were aligned using Clustal W (www.ebi.ac.uk/clustalw; Thompson *et al*., 1994), corrected by eye and adjusted. The alignment of ITS-2 sequences was further improved according to a secondary structure model (Chilton *et al*., 1998; Newton *et al*., 1998) to increase positional similarity in regions with greater variation among species. For each unique sequence obtained, the number of samples with that particular SSCP profile was used to obtain the total number of sequences following SSCP (‘Sequences examined following SSCP’ in Table 2). Unique new sequences have been deposited in GenBank (accession numbers PP919566–PP919604).

A phylogenetic tree was constructed by concatenating the ITS1 and ITS2 sequence data, using *Arundelia dissimilis* as an outgroup. Sequences were aligned using MUSCLE (Edgar, 2004), followed by manual adjustment using Mesquite v.3.61 (Maddison and Maddison, 2015). Phylogenetic analyses of the data were conducted by Bayesian inference (BI) using MrBayes v.3.2.6 (Ronquist *et al*., 2012), with the likelihood parameters based on the Akaike Information Criteria test in IQ-TREE v.2 (Minh *et al*., 2020). Specifically, the number of substitutions (Nst) was set at two, and a gamma-distribution was used. Posterior probability (pp) values were calculated from 2 000 000 generations using four simultaneous tree-building chains, with trees being saved every 100th generation. The standard deviation of split frequencies was <0.01, and the potential scale reduction factor approached one, indicating convergence. To ensure convergence and insensitivity to priors, the analyses were run three times. Finally, a 50% majority rule consensus tree was constructed based on the final 75% of trees generated by BI.

Pairwise distances (percentage identity) were generated using Geneious 2024.0.7.(https://www.geneious.com) (Supplementary File 1).

## Results

**Figure 1.**
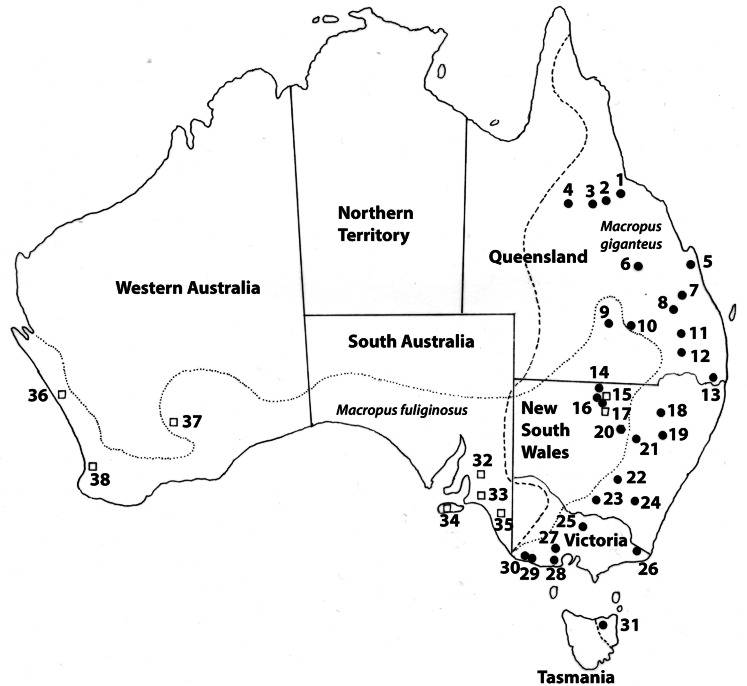
Localities at which specimens of *Cloacina* spp. were collected from *Macropus fuliginosus* (open squares) and *M. giganteus* (closed circles) for molecular studies. Locality numbers: Queensland: 1, Homestead; 2, Prairie; 3, Hughenden; 4, Nelia; 5, Rockhampton; 6, Bogantungen; 7, Theodore; 8, Taroom; 9, Charleville; 10, Mungallalla; 11, Miles; 12, Moonie; 13, Killarny; New South Wales: 14, Enngonia; 15, Bourke (both kangaroo species collected at this site); 16, Byrock; 17, Glenariff; 18, Narrabri; 19, Coonabarabran; 20, Nyngan; 21, Trangie; 22, West Wyalong; 23, Beckom; 24, Bondo State Forest; Victoria: 25, Nagambie; 26, Genoa; 27, Bacchus Marsh; 28, Avalon; 29, Portland; 30, Cape Bridgewater; 31, Evandale; South Australia: 32, Kersbrook; 33, Ashbourne; 34, Kangaroo Island; 35, Tintinara; Western Australia: 36, Geraldton; 37, Kalgoorlie; 38, Waroona. Dotted lines indicate the geographical distributions of *Macropus fuliginosus* and *M. giganteus*.

All collection localities, together with their coordinates are provided in Table 1 and are shown in Fig. 1. Distribution maps of different genotypes within each species of *Cloacina* examined are not provided for species with distinct genotypes in *M. fuliginosus* and *M. giganteus* (*C. hera*, *C. hermes*, *C. hestia*) following host species boundaries as these data are available in Table 1. Distribution maps of genotypes are provided (Figs 3–6) for species (*C. artemis*, *C. expansa*, *C. obtusa*, *C. selene*) in which nematode genotypes do not correspond with host species boundaries.

**Table 1.** Collection localities and the number of various *Cloacina* species examined at each locality.

Cloacina sp.		C. artemis	C. expansa	C. herceus	C. hera	C. hermes	C. hestia	C. magnipapillata	C. obtusa	C. selene
Locality	Coordinates									
Queensland										
Homestead ^1^	20°22′ S 145°39′ E		2	4				4		
Nelia^4^	20°39′ S 142°13′ E		4	2				4		
Hughenden^3^	20°51′ S 144°12′ E			3				5		
Prairie^2^	20°52′ S 144°36′ E		6	7				8		
Rockhampton^5^	23°01′ S 150°16′ E	5	5	5				5	4	
Bogantungen^6^	23°39′ S 147°18′ E		4					2	7	
Theodore^7^	24°57′ S 150°05′ E		2	7				7		
Taroom^8^	25°57′ S 149°53′ E		3	5			3	1	3	
Charleville^9^	26°20′ S 146°17′ E		5	7	1			1	6	
Mungallalla^10^	26°26′ S 147°22′ E		4	4	1		5			
Miles^11^	26°40′ S 150°01′ E		1					4		
Moonie^12^	27°43′ S 150°22′ E		3	4						
Killarney^13^	28°20′ S 152°18′ E								4	
New South Wales										
Enngonia^14^	29°19′ S 145°51′ E		7		2			3	4	
Bourke ^15^	30°05′ S 145°56′ E	(3)	(9)	4			2 (9)	5 (6)	2 (7)	
Narrabri^18^	30°20′ S 149°47′ E				5				4	
Byrock^16^	30°40′ S 146°24′ E		(4)	(7)			(2)		(8)	
Glenariff^17^	30°49′ S 146°33′ E						(3)			
Coonabarabran^19^	31°16′ S 149°17′ E			2	6	5	1		4	
Nyngan^20^	31°34′ S 147°12′ E		3		1			1		
Trangie^21^	32°02′ S 147°59′ E		1	8					4	
West Wyalong^22^	33°55′ S 147°12′ E		1			3			6	
Beckom^23^	34°20′ S 146°58′ E			3	1	7			4	1
Bondo S F^a^^24^	35°25′ S 148°45′ E			1	4				3	7
Victoria										
Nagambie^25^	36°47′ S 145°10′ E	9	10	11		1	5	7	5	5
Genoa^26^	37°32′ S 149°38′ E								5	1
Bacchus Marsh^27^	37°41′ S 144°26′ E		4	2					4	6
Avalon^28^	38°02′ S 144°28′ E			4						8
Portland^29^	38°21′ S 141°36′ E	2	1	4	6	5			5	7
C. Bridgewater^30^	38°24′ S 141°25′ E	1	4							
Tasmania										
Evandale^31^	41°34′ S 147°13′ E								9	7
South Australia										
Kersbrook^32^	34°47′ S 138°51′ E					(10)		(4)	(3)	
Ashbourne^33^	35°17′ S 138°46′ E		(6)			(6)		(7)	(16)	
Kangaroo Is.^34^	35°50′ S 138°03′ E	(1)				(13)		(27)	(4)	(7)
Tintinara^35^	35°53′ S 140°04′ E	(2)	(2)						(5)	(5)
Western Australia										
Geraldton^36^	28°46′ S 114°37′ E		(6)					(6)	(6)	
Kalgoorlie^37^	30°45′ S 121°28′ E	(4)	(5)		(5)	(6)		(3)	(6)	
Waroona^38^	32°51′ S 115°59′ E	(3)	(6)				(5)	(6)	(6)	(7)
**Total**		30	108	94	32	56	35	116	144	61

Numbers in parentheses represent nematode specimens from *M. fuliginosus*; those not within parentheses are from *M. giganteus.*

Locality superscript numerals correspond with those in Fig. 1.

a State Forest.

The number of specimens of each nematode species used, the numbers of ITS-1 and ITS-2 sequences obtained representing all SSCP profiles and all localities, the numbers of different genotypes encountered and the base pair numbers at which differences were noted are shown in Table 2.

**Table 2.** Summary of sequence differences between eastern and western genotypes of *Cloacina* spp

Cloacina artemis																								
Sequences examined following SSCP: M. fuliginosus: 10 ITS-1, 12 1TS-2; M. giganteus: 17 ITS-1, 17 ITS-2																								
	Genotype 1						Genotype 2						Genotype 3						Genotype 4					
GenBank codes	PP919566 PP919585						PP919567 PP919586						− PP919587						PP919568 PP919588					
	ITS-1	ITS-2					ITS-1	ITS-2					ITS-1	ITS-2					ITS-1	ITS-2				
Base-pair number	374	48	58	110	143	171	374	48	58	110	143	171	374	48	58	110	143	171	374	48	58	110	143	171
Consensus genotype	**A**	**C**	**T**	**A**	**T**	**G**	**G**	**T**	**C**	**T**	**T**	**G**	**A**	**T**	**C**	**A**	**C**	**G**	**A**	**T**	**C**	**A**	**C**	**C**
Number of base pair differences: ITS-1: 1; ITS-2: 5; Number of base-pairs: ITS = 1, 387; ITS-2, 226																								

Cloacina expansa				
Sequences examined following SSCP: M. fuliginosus: 36 ITS-1, 38 1TS-2; M. giganteus: 64 ITS-1, 66 ITS-2				
	Genotype 1		Genotype 2	
GenBank codes	PP919588 PP919589		PP919570 PP919590	
	ITS1	ITS2	ITS1	ITS2
Base-pair number	198	-	198	-
Consensus genotype	**G**	-	**A**	-
Number of base pair differences: ITS-1: 1; ITS-2: 0 Number of base pairs: ITS-1, 378; ITS-2, 219				

Cloacina hera																										
Sequences examined following SSCP: M. fuliginosus: 5 ITS-1, 5 1TS-2; M. giganteus: 23 ITS-1, 20 ITS-2																										
	Genotype 1													Genotype 2												
GenBank codes	PP919571 PP919591													PP919591 PP919592												
	ITS-1						ITS-2							ITS-1						ITS-2						
Base-pair number	45	102	107	165	170	175	57	84	85	116	120	124	191	45	102	107	165	170	175	57	84	85	116	120	124	191
Consensus genotype	**A**	**A**	**T**	**C**	**G**	**T**	**T**	**T**	**C**	**A**	**A**	**G**	**G**	**G**	**G**	**C**	**T**	**A**	**C**	**C**	**C**	**T**	**G**	**G**	**A**	**C**
Base-pair number	202	209	256	309	342	366								202	209	256	309	342	366							
Consensus genotype	**G**	**T**	**T**	**G**	**A**	**A**								**A**	**A**	**C**	**G**	**G**	**G**							
Number of base pair differences: ITS-1: 12; ITS-2: 7; Number of base pairs: ITS-1, 337; ITS-2, 220																										

Cloacina hermes																								
Sequences examined following SSCP: M. fuliginosus: 20 ITS-1, 20 1TS-2; M. giganteus: 11 ITS-1, 11 ITS-2																								
	Genotype 1																							
GenBank codes	PP919574 PP919594																							
	ITS-1															ITS-2								
Base-pair number	22	47	49	51	56	81	95	101	107	109	123	161	178	179	199	37	96	106	109	111	119	124	129	133
Consensus genotype	**C**	**A**	**A**	**A**	**A**	**A**	**G**	**G**	**C**	**T**	**T**	**G**	**A**	**T**	**T**	**T**	**C**	**T**	**T**	**T**	**G**	**A**	**A**	**A**
Base-pair number	200	228	231	273	309	311	316	323	345	370						143	147	177	178	180	192	193	194	208
Consensus genotype	**G**	**T**	**C**	**G**	**T**	**A**	**A**	**A**	**A**	**T**						**T**	**A**	**A**	**G**	**A**	**A**	**A**	**A**	**A**

	Genotype 2																							
GenBank codes	PP919575 PP919595																							
	ITS-1															ITS-2								
Base-pair number	22	47	49	51	56	81	95	101	107	109	123	161	178	179	199	37	96	106	109	111	119	124	129	133
Consensus genotype	**T**	**A**	**T**	**G**	**G**	**G**	**C**	**A**	**T**	**G**	**A**	**T**	**T**	**C**	**C**	**G**	**T**	**C**	**C**	**C**	**T**	**G**	**G**	**T**
Base-pair number	200	228	231	273	309	311	316	323	345	370						143	147	177	178	180	192	193	194	208
Consensus genotype	**A**	**A**	**T**	**A**	**C**	**T**	**G**	**T**	**G**	**G**						**C**	**G**	**G**	**T**	**T**	**T**	**T**	**T**	**G**
Number of base pair differences: ITS-1: 24; ITS-2: 18; Number of base pairs: ITS-1, 377; ITS-2, 223																								

Cloacina hestia																						
Sequences examined following SSCP: M. fuliginosus: 20 ITS-1, 14 1TS-2; M. giganteus: 13 ITS-1, 2 ITS-2																						
	Genotype 1																					
GenBank codes	PP919576 PP919596																					
	ITS-1															ITS-2						
Base-pair number	38	45	75	105	118	135	200	201	209	296	339	342	346	348	358	96	109	124	129	138	215	232
Consensus genotype	**A**	**G**	**T**	**T**	**C**	**T**	**C**	**A**	**C**	**C**	**T**	**G**	**G**	**A**	**A**	**C**	**C**	**A**	**A**	**G**	**G**	**C**

	Genotype 2																					
GenBank codes	PP919577 PP919597																					
	ITS-1															ITS-2						
Base-pair number	38	45	75	105	118	135	200	201	209	296	339	342	346	348	358	96	109	124	129	138	215	232
Consensus genotype	**G**	**A**	**C**	**G**	**G**	**A**	**T**	**G**	**T**	**T**	**A**	**A**	**A**	**C**	**T**	**C**	**C**	**A**	**A**	**G**	**G**	**C**
Number of pair differences: ITS-1: 15; ITS-2: 8; Number of base pairs: ITS-1, 377; ITS-2, 222																						

Cloacina magnipapillata																						
Sequences examined following SSCP: M. fuliginosus: 53 ITS-1, 55 1TS-2; M. giganteus: 31 ITS-1, 44 ITS-2																						
	Genotype 1											Genotype 2										
GenBank codes	PP919578 PP919598											PP919579 PP919599										
	ITS-1							ITS-2				ITS-1							ITS-2			
Base-pair number	32	53	234	245	293	308	332	77	119	183	186	32	53	234	245	293	308	332	77	119	183	186
Consensus genotype	**A**	**A**	**A**	**T**	**C**	**A**	**A**	**T**	**T**	**T**	**G**	**C**	**G**	**G**	**G**	**T**	**G**	**C**	**C**	**C**	**G**	**A**
Number of base pair differences: ITS-1: 7; ITS-2: 4; Number of base pairs: ITS-1, 376; ITS-2, 225																						

Cloacina obtusa																		
Sequences examined following SSCP: M. fuliginosus: 59 ITS-1, 56 1TS-2; M. giganteus: 73 ITS-1, 75 ITS-2																		
	Genotype 1						Genotype 2						Genotype 3					
GenBank codes	PP919580 PP919600						PP919581 PP919601						PP919582 PP919602					
	ITS-1		ITS-2				ITS-1		ITS-2				ITS-1		ITS-2			
Base-pair number	210	325	57	89	181	190	210	325	57	89	181	190	210	325	57	89	181	190
Consensus genotype	**T**	**C**	**T**	**T**	**T**	**C**	**A**	**A**	**C**	**C**	**C**	**T**	**A**	**A**	**C**	**C**	**T**	**C**
Number of base pair differences: ITS-1: 2; ITS-2: 5; Number of base pairs: ITS-1, 378; ITS-2, 222																		

Cloacina selene																		
Sequences examined following SSCP: M. fuliginosus: 12 ITS-1, 14 1TS-2; M. giganteus: 29 ITS-1, 38 ITS-2																		
	Genotype 1									Genotype 2								
GenBank codes	PP919583 PP919603									PP919584 PP919604								
	ITS-1					ITS-2				ITS-1					ITS-2			
Base-pair number	59	95	97	157	330	3	29	92	174	59	95	97	157	330	3	29	92	174
Consensus genotype	**T**	**G**	**A**	**A**	**A**	**T**	**T**	**T**	**T**	**C**	**A**	**G**	**G**	**C**	**C**	**C**	**G**	**A**
Number of base pair differences: ITS-1: 5; ITS-2: 4; Number of base pairs: ITS-1, 377; ITS-2, 224																		

**Figure 2.**
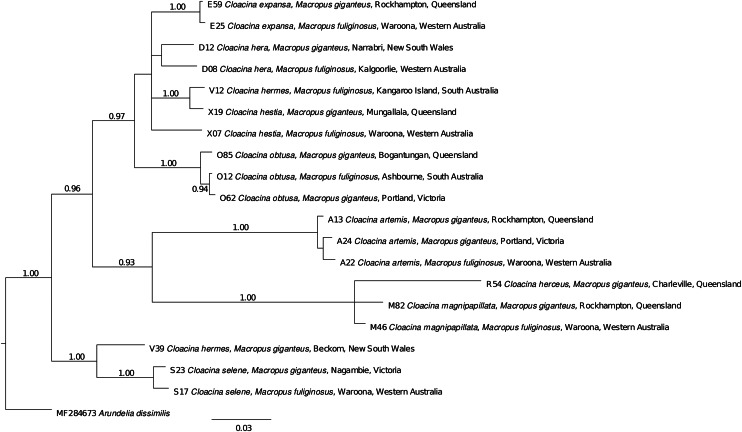
Phylogenetic analysis of concatenated ITS1 and ITS2 sequence data to infer the relationships of *Cloacina* species. The tree was constructed using Bayesian Inference method (MrBayes) and used *Arundelia dissimilis* as an outgroup. Posterior probabilities less than 0.90% are not displayed. The scale bar represents the number of substitutions per site.

The phylogenetic analysis (Fig. 2) indicated that the different genotypes of *C. artemis, C. expansa, C, hera, C. hestia, C. magnipapillata, C. obtusa* and *C. selene* found in *M. fuliginosus* and *M. giganteus* each formed separate clades although not necessarily restricted to a single host species. By contrast, specimens of *C. hermes* occurred in 2 highly divergent clades, one, found in *M. fuliginosus*, associated with *C. hestia* and the other, in *M. giganteus*, associated with C. selene.

Pairwise distances generated using Geneious are presented in Supplementary File 1. The supplementary material for this article can be found at [DOI].

### Cloacina artemis

**Figure 3.**
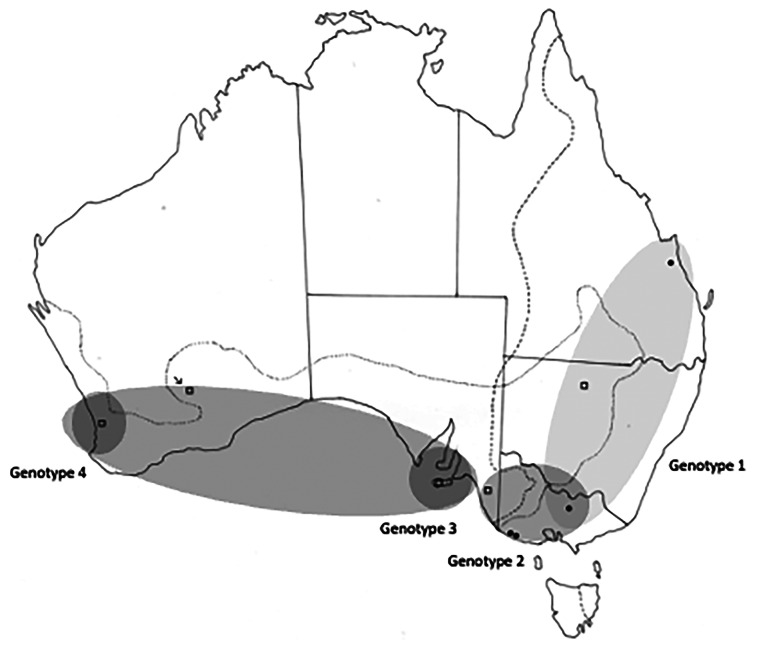
Collection sites for *Cloacina artemis*, showing the distribution of the four different genotypes identified. Solid circles indicate specimens collected from *M. giganteus*; open squares indicate specimens collected from *M. fuliginosus*. The arrow indicates specimens collected from *M. fuliginosus*, initially identified as *C. artemis*, but now identified as a closely related but apparently un-named new species. Dotted lines indicate the geographical distributions of *Macropus fuliginosus* and *M. giganteus*.

A total of 30 specimens of *C. artemis* were examined (Table 1). Four genotypes were detected, in some cases with overlapping distributions (Table 2, Fig. 3). Genotype 1 was found in *M. giganteus* at Rockhampton, Qld (location 5) and Nagambie in Vic (location 25), and in *M. fuliginosus* in the zone of host sympatry at Bourke, NSW (Location 15). A second genotype occurred in *M. giganteus* in Vic at Nagambie and the Portland/Cape Bridgewater region (locations 29, 30) as well as in *M. fuliginosus* in an adjacent area in the south-east of SA (location 35). A third genotype was present in *M. fuliginosus* on Kangaroo Island, SA (location 34) with a minor variant of it differing at only one base pair in WA (location 38), here treated as a fourth genotype (Fig. 3). Specimens from Kalgoorlie, WA (locality 37), initially identified morphologically as *C. artemis*, proved to be a closely related but currently undescribed species (Fig. 3) deposited in SAM (45578) which warrants additional study.

### Cloacina expansa

**Figure 4.**
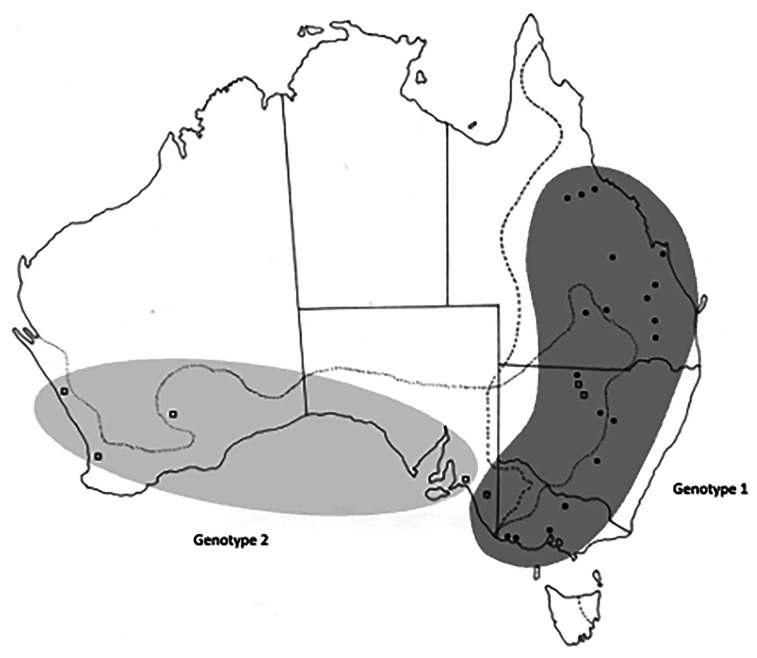
Collection sites for *Cloacina expansa*, showing the distribution of the two different genotypes identified. Solid circles indicate specimens collected from *M. giganteus*; open squares indicate specimens collected from *M. fuliginosus*. Dotted lines indicate the geographical distributions of *Macropus fuliginosus* and *M. giganteus*.

A total of 94 specimens of *C. expansa* were examined (Table 1). Variation in the sequences was limited. In ITS-1, all specimens from *M. giganteus* displayed a G at base-pair (bp) 198, while all specimens from *M. fuliginosus* exhibited a transition to A at the same position, apart from two specimens from Tintinara in SA (location 35) which retained a G. The latter was the only collection of *C. expansa* from *M. fuliginosus* near the zone of host sympatry. Two genotypes were recognised, one primarily in *M. giganteus* and in a single *M. fuliginosus* near the zone of kangaroo sympatry and a second genotype in *M. fuliginosus* beyond the zone of sympatry (Fig. 4).

### Cloacina hera

A total of 32 specimens of *C. hera* were examined (Table 1). All specimens from *M. giganteus* from Charleville and Mungallalla in Qld (localities 9, 10), Enngonia, Narrabri, Coonabarabran, Beckom, Bondo State Forest in NSW (localities 14, 18, 19, 23, 24) and Portland in Vic (locality 29) exhibited the same genotype. Specimens from a single locality in WA (Kalgoorlie) (locality 37) differed at 12 bp in ITS-1 and seven bp in ITS-2 (Table 2). The two genotypes detected were each found in a single host species.

### Cloacina herceus

A total of 94 specimens of *C. herceus* were examined using SSCP (Table 1). ITS-1 and ITS-2 sequences were subsequently obtained from a total of 20 and 30 nematodes respectively from *M. giganteus*, including specimens from the type locality, Coonabarabran, NSW, and from one *M. fuliginosus,* representing all SSCP profiles. Variation within both internal transcribed spacers was limited to a single transversion in ITS-1 (T-G) at bp 340 in a single specimen. Only a single collection of this species was obtained from *M. fuliginosus* in the zone of kangaroo sympatry with *M. giganteus* in north-western NSW and all 7 nematodes from this kangaroo exhibited the same consensus sequence (not shown) as that of specimens from *M. giganteus* (GenBank accession numbers PP919573, PP919593).

### Cloacina hermes

A total of 56 specimens of *C. hermes* were examined (Table 1). There was a single genotype in nematodes from *M. fuliginosus* from Kersbrook, Ashbourne, and Kangaroo Island in SA (localities 32, 33, 34) and Kalgoorlie in WA (locality 37) differing from that in nematodes from *M. giganteus* from Coonabarabran, West Wyalong and Beckom in NSW (localities 19, 22, 23) at 24 ITS-1 and 18 ITS-2 bp (Table 2), with no samples of this species collected in a zone of host sympatry. Due to the large numbers of bp differences, all specimens were re-examined morphologically, but no differences were noted between specimens from the two species of kangaroos. The two genotypes did not form a single clade in the phylogenetic analysis (Fig. 2), with the specimens from *M. fuliginosus* included in the *C. hestia* clade and those from *M. giganteus* in the *C. selene* clade.

### Cloacina hestia

A total of 35 specimens of *C. hestia* were examined (Table 1). Within specimens from *M. giganteus*, collected at Taroom and Charleville in Qld (localities 8, 9), Bourke and Coonabarabran in NSW (localities 15 and 19) and Nagambie in Vic (locality, 25), in the ITS-1 sequences there was a single transversion from T to G in one specimen from Coonabarabran. Specimens from *M. fuliginosus* collected in the zone of kangaroo sympatry at Bourke, Byrock and Glenariff in NSW (localities 15, 16, 17) exhibited sequences identical to those found in *M. giganteus* in the same area. Specimens from *M. fuliginosus* collected outside the zone of sympatry at Waroona, WA (locality, 38), differed consistently at 15 ITS-1 and eight ITS-2 bp respectively (Table 2).

### Cloacina magnipapillata

A total of 116 specimens of *C. magnipapillata* were examined (Table 1). ITS sequences in specimens from *M. giganteus* in Qld, NSW and Vic were identical (localities 1–6, 9, 14, 15, 25) as were those from *M. fuliginosus* in the zone of kangaroo sympatry (locality 15). Those from *M. fuliginosus* in SA and WA (localities 32–38), beyond the zone of sympatry, differed consistently at 7 ITS-1 bp and 4 ITS-2 bp (Table 2).

### Cloacina obtusa

**Figure 5.**
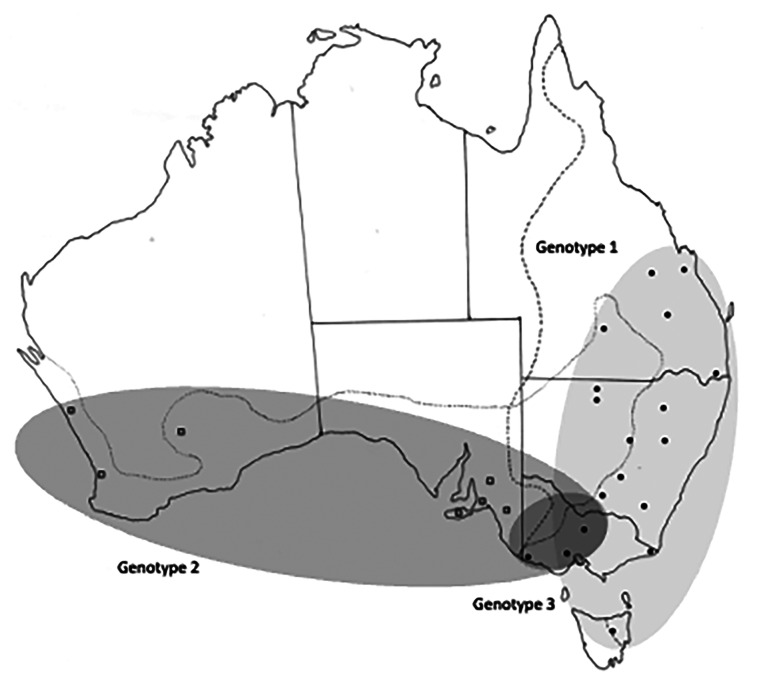
Collection sites for *Cloacina obtusa*, showing the distribution of the three different genotypes identified. Solid circles indicate specimens collected from *M. giganteus*; open squares indicate specimens collected from *M. fuliginosus*. Dotted lines indicate the geographical distributions of *Macropus fuliginosus* and *M. giganteus*.

A total of 144 specimens of *C. obtusa* were examined (Table 1). In ITS-1 sequences, the T and C bases at positions 210 and 325 respectively in specimens from *M. giganteus* from Qld (localities 5, 6, 8, 9, 13), NSW (localities 14, 16, 18, 19, 21–24) and eastern Vic (locality 26) and from *M. fuliginosus* in NSW (localities 15, 16), were replaced by an A, in specimens from *M. giganteus* from central and western Vic, Nagambie, Bacchus Marsh, Portland (localities 25, 27, 29) and from *M. fuliginosus* in SA and WA (localities 32–38) (Table 2). In ITS-2 sequences, the T in positions 57, 89 and 206 in specimens from *M. giganteus* from Qld (localities 5, 6, 8, 9, 13), NSW (localities 14, 16, 18, 19, 21–24) and eastern Vic (locality 26), as well as from *M. fuliginosus* in NSW (localities 15, 16), were replaced by a C in specimens from *M. giganteus* in central and western Victorian (localities 25, 27, 29) and from *M. fuliginosus* in SA and WA (localities 32–38). In addition, the C in position 181 of the consensus sequence was replaced by a T in specimens from central and southern Vic (Nagambie, Bacchus Marsh, Portland, localities 25, 27, 29). Consequently, three genotypes were recognised, the first primarily in *M. giganteus*, but also in *M. fuliginosus* in areas of sympatry in north-western NSW; the second in *M. fuliginosus* beyond the zone of sympatry and a third in *M. giganteus* in south-western Vic (localities 25, 27, 29) (Fig. 5). In the latter cluster, the ITS sequences differed at only one base pair from those in *M. fuliginosus* (Table 2).

### Cloacina selene

**Figure 6.**
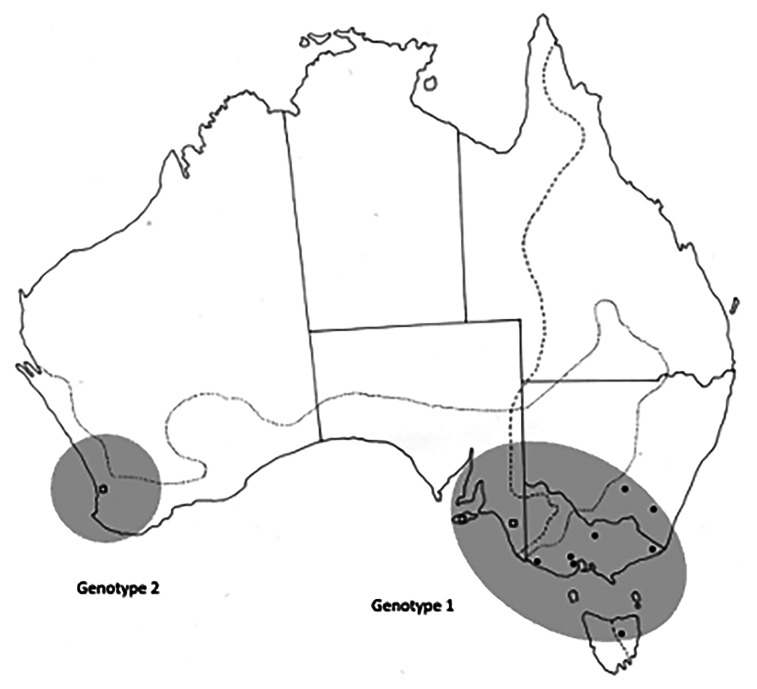
Collection sites for *Cloacina selene*, showing the distribution of the two different genotypes identified. Solid circles indicate specimens collected from *M. giganteus*; open squares indicate specimens collected from *M. fuliginosus*. Dotted lines indicate the geographical distributions of *Macropus fuliginosus* and *M. giganteus*.

A total of 61 specimens of *C. selene* were examined (Table 1). ITS sequences of specimens from *M. giganteus* from NSW (localities 23, 24), Vic (localities 25–29) and Tas (locality 31) as well as from *M. fuliginosus* in SA (localities 34, 35) were identical genetically (Table 2). Specimens from *M. fuliginosus* from WA (locality 38) differed from all other specimens consistently at 5 ITS-1 and 4 ITS-2 bp (Table 2, Fig. 6).

## Discussion

The choice of ITS-1 and ITS-2 as molecular markers in this study was deliberate as they are relatively conservative indicators of genetic change in strongylid nematodes and any changes detected are more likely to indicate differentiation at the species level than the more highly variable cytoplasmic genes (e.g. Dame *et al*., 1993). Since our hypothesis was that speciation in the grey kangaroos may have initiated speciation in their nematodes, the use of genetic markers which were likely to indicate speciation in the parasites was considered more appropriate.

In most of the nematode species examined, genetic differences were detected between populations from eastern and western grey kangaroos. However, the extent of the genetic differences varied markedly between different nematode species as did the geographical boundaries between the eastern and western nematode genetic populations.

Sample sizes of different nematode species varied greatly and were influenced by the different prevalences, abundances and geographical distributions of the nematode species at collection sites (Beveridge, 2023). A potential additional bias is that it is more likely that larger species (e.g. *C. expansa*, *C. obtusa*, 10–19 mm) rather than the smaller species (e.g. *C. artemis*, *C. selene*, 6–10 mm) (Beveridge, 1998) were collected. Nevertheless, sequences were obtained from more than 80 individual nematodes in the cases of *C. expansa*, *C. herceus*, *C. magnipapillata* and *C. obtusa*, compared with numbers in the 20s in the cases of *C. artemis*, *C. hera* and *C. hestia* (Table 1). Fewer localities were examined in Western Australia and only a single locality in Tasmania (Table 1). These limitations need to be taken into consideration when interpreting the data.

*Cloacina herceus* was included even though it is primarily a parasite of *M. giganteus* and only occurs in *M. fuliginosus* in the zone of kangaroo sympatry (Beveridge, 1998). *Cloacina herceus* occurs across a wide geographic range, including an insular population of *M. giganteus* in Tasmania. The virtual absence of genetic variation in *C. herceus* over a sampling distance of some 2000 km suggests that an extensive geographic distribution alone has not led to significant genetic differentiation in this parasite and is consistent with similar results obtained from species of *Cloacina* found in *Wallabia bicolor* and *Osphranter robustus* (Shuttleworth *et al*., 2014, 2016). A phylogeographic study of *M. giganteus* revealed little genetic differentiation within the host species, including the insular population in Tasmania (Zenger *et al*., 2003). These authors did however identify some differences between northern and southern kangaroo populations and suggested that the northern populations were more recent and derived from a relatively small number of southern founders. Examination of *C. herceus* from *M. fuliginosus* in the zone of kangaroo sympatry indicated an apparent instance of host switching with no apparent genetic differentiation of the nematodes in this kangaroo host.

In each of the remaining nematode species with both eastern and western populations, genetic differences were detected. However, the extent of the genetic differences varied considerably between nematode species as well as the geographical distribution of particular genotypes generally and with respect to the zone of host sympatry.

Minimal genetic differences were seen in specimens of *C. expansa,* with identical ITS-2 sequences in all specimens examined and with a single base change in ITS-1 between eastern and western populations. The latter change was consistent in the samples examined but was not entirely aligned with host species, with the eastern genotype being found in *M. fuliginosus* as far west as Tintinara, South Australia, some 100 km west of the currently known limit of the distribution of *M. giganteus* (Fig. 4). In the zone of kangaroo sympatry in New South Wales, specimens collected from *M. fuliginosus* were of the eastern genotype and it was concluded in this instance, as with *C. herceus*, that host switching had probably occurred. However, the number of specimens examined in the zone of kangaroo sympatry was small and further sampling is required to confirm this result.

More extensive genetic variation was seen in the case of *C. magnipapillata*, with consistent differences at seven ITS-1 bp and four ITS-2 bp between eastern and western populations. In this case, the geographical distributions of the eastern and western genetic types corresponded with that of the host species. In the zone of kangaroo sympatry in New South Wales, specimens collected from *M. fuliginosus* were of the eastern genotype and it was concluded in this instance, again as with *C. herceus*, that host switching had occurred. However, the number of specimens examined in the zone of sympatry from this species was also small and further sampling is required to confirm this result. Further south in Victoria and Tasmania, *C. magnipapillata* is replaced by *C. pelops* and this species does not occur in *M. fuliginosus* (Beveridge, 1998). Beveridge (1998) noted slight morphological differences between eastern and western populations of *C. magnipapillata* (spicule lengths), a finding consistent with the genetic data.

Substantial sequence differences were noted between eastern and western populations of *C. hestia* (Table 2). The two differing genotypes corresponded with the two different host species. Re-examination of specimens of *C. hestia* from the south-west of Western Australia indicated that the mean length of their spicules was shorter than those of other populations (unpublished observations), thus potentially providing some morphological support for the genetic differences noted.

*Cloacina hera* also exhibited substantial genetic differences between eastern and western populations of nematodes (Table 2) and thus the results are similar to those found in several other species. However, the number of localities sampled for this species was too limited to allow firm conclusions to be drawn regarding the geographical distributions of the two genotypes.

In the case of *C. artemis*, both eastern and western populations were subdivided based on a single base pair difference into northern and southern populations in the case of the eastern genotype and between the Kangaroo Island (in South Australia) and Western Australian populations in the case of the western genotype (Fig. 3). Given the small number of nematodes examined, these differences warrant further investigation. Neaves *et al*. (2009) reported genetic differences between western (south-west of Western Australia), central (Nullarbor region), eastern (east of the Flinders Ranges) and Kangaroo Island populations of *M. fuliginosus*, which may be reflected in the genetic differences seen here between the nematode populations from Kangaroo Island and Western Australia. However, no similar genetic differences are known within *M. giganteus* (Zenger *et al*., 2003) and at one locality in Victoria (Nagambie), the two eastern genotypes overlapped.

Populations of *C. selene* were represented by two genotypes which did not correspond with their host species. The eastern population in *M. giganteus* in Victoria and Tasmania extended into the south-east of South Australia and on to Kangaroo Island in *M. fuliginosus*, differing from the population in *M. fuliginosus* in Western Australia (Fig. 6). Some caution is also needed in interpreting these data as only a single locality was examined in Western Australia. There are several possible reasons for a lack of concordance in some instances between host species and parasite genotype in south-eastern Australia. The zone of contact between the two host species is known to have changed over time and this is particularly evident in the westward expansion of *M. giganteus* in recent decades (Caughley *et al*., 1984). *Macropus giganteus* was present on Kangaroo Island and therefore also presumably in the adjacent Adelaide region in the late Pleistocene to mid-Holocene (Seersholm *et al*., 2021) and its distribution has since retracted eastwards. This may explain the current occurrence of the eastern genotype of *C. selene* in *M. fuliginosus* in South Australia. The occurrence of the eastern genotype of *C. artemis* and *C. expansa* in a single *M. fuliginosus* from the south-east of South Australia could also be accounted for by the same hypothesis. However, *M. giganteus* is now common in the south-east of South Australia, south of Naracoorte (Moloney *et al*., 2021) and therefore, it is equally possible that the single *M. fuliginosus* examined had been in contact with populations of *M. giganteus* further to the south as the precise distribution of the two species of kangaroos in this area is not known. A further possibility is that once a nematode genotype has switched hosts, it may continue to disperse within the new host species beyond the actual zone of sympatry.

*Cloacina obtusa* also exhibited distinct eastern and western genotypes (Table 2). The eastern genotype of *C. obtusa* occurred in *M. giganteus* and in *M. fuliginosus* in the zone of sympatry in north-western New South Wales. The western genotype was found in *M. fuliginosus* beyond the zone of host sympatry. However, a third genotype within *C. obtusa* was detected in *M. giganteus*, apparently limited to an area in western Victoria. This genotype differs from the western genotype at only one base pair in ITS-2 (Fig. 5). This geographical region is adjacent to but not within the current zone of host sympatry. The occurrence of a genotype of *C. obtusa* in *M. giganteus* in the south-west of Victoria, similar, but not identical to that found in *M. fuliginosus* could possibly be explained if the latter kangaroo species formerly extended further eastwards into western Victoria, leaving behind nematode genotypes as the host distribution altered (genetic introgression). It could equally be explained by contact and transfer of *C. obtusa* from *M. fuliginosus* to *M.giganteus* and the subsequent spread of the genotype within south-western populations of *M. giganteus*. Both species of kangaroo occur in sympatry in the lower Glenelg region of south-western Victoria, close to Cape Bridgewater and Portland (Bennett, 1995*a*, 1995*b*), but the genotype extends across Victoria as far as Nagambie, north of Melbourne, some 750 km away. Unfortunately, nothing is known of the former host ranges of the two kangaroo species in this area to distinguish between these hypotheses.

In the case of *C. hermes*, the nematodes from *M. fuliginosus* differed from those in *M. giganteus* at 42 base pairs and in the phylogenetic analysis (Fig. 2), the two populations of nematodes were aligned with *C. hestia* for the specimens examined from *M. fuliginosus* and with *C. selene* for those from *M. giganteus*, suggesting an independent evolutionary origin of these genotypes. The current molecular data therefore suggest that *C. hermes*, as currently defined morphologically, may consist of two phylogenetically unrelated species. Consequently, a morphological re-examination of this species appears to be warranted.

The hypothesis examined in this study, that the recent speciation of two closely related kangaroo host species should result in genetic differentiation in their parasites was supported by the finding of varying levels of genetic differentiation in eastern and western populations of *C. artemis*, *C. expansa*, *C. hera*, *C. hestia*, *C. magnipapillata* and *C. obtusa*. What was not predicted was the differing extent of genetic differentiation between the various nematode species, ranging from a single base pair in *C. expansa* to extensive differences between populations of *C. hera*, *C. magnipapillata* and *C. obtuse* The differing phylogenetic associations of the genotypes of *C. hermes* was unexpected. No claim is made here that the different genotype pairs represent distinct species as, according to Nadler (2002), levels of genetic difference do not necessarily provide reliable indicators of separate species. However, further investigation using additional markers (Nadler and Pérez-Ponce de León, 2011) may identify a number of cryptic species pairs in eastern and western grey kangaroos.

Samples of *C. artemis*, *C. hera, C. hestia* and *C. selene* were small and provide limited information on the geographical distribution of the genotypes identified, but in the cases of *C. expansa*, *C. magnipapillata* and *C. obtusa,* more extensive sampling has provided greater detail. The current findings that populations of *C. hermes* in *M. fuliginosus* and *M. giganteus* are phylogenetically distinct suggests that not all of the species studied may exhibit host related vicariance and that each nematode species needs to be assessed individually.

The distribution of genotypes in zones of host sympatry or even areas in close proximity to them appears to be complex and, in the case of *C. obtusa*, the distinctive genotype in western Victoria, close to a zone of sympatry warrants further genetic studies.

While this study has provided some evidence that host speciation of the two grey kangaroo species appears to have at least been associated with genetic diversification in some of its parasite species, why this has not been translated to a broader scale of co-speciation between species of *Cloacina* and their macropodid hosts in which the principal mode of speciation appears to be by colonisation (Chilton *et al*., 2017) remains to be investigated. Additional studies are needed to determine how widespread the phenomenon of potential speciation reported here in grey kangaroos may occur in other closely related macropodid host species.

## Supporting information

Middleton et al. supplementary material Middleton et al. supplementary material

## Data Availability

No data available.

## Supplementary material

The supplementary material for this article can be found at https://doi.org/10.1017/S0031182024001458
